# Development of an online optical prototype for the simultaneous analysis of defects or gels in industrially synthesized polypropylene films. Part 1: Comparison with ASTM D 3351–93 methods

**DOI:** 10.1016/j.mex.2024.102688

**Published:** 2024-04-01

**Authors:** Hernández-Fernández Joaquin, Ortiz Katherine, Lopez-Martinez Juan

**Affiliations:** a Chemistry Program, Department of Natural and Exact Sciences, San Pablo Campus, University of Cartagena, Cartagena 130015, Colombia; b Chemical Engineering Program, School of Engineering, Universidad Tecnológica de Bolivar, Parque Industrial y Tecnológico Carlos Vélez Pombo Km 1 Vía Turbaco, Cartagena 130001, Colombia; c Department of Natural and Exact Science, Universidad de la Costa, Barranquilla 080002, Colombia; d Centro de investigación e invención en ciencias e ingeniería, CECOPAT & A, Cartagena, Colombia; e Institute of Materials Technology (ITM), Plaza Ferrandiz ‘ and Carbonell s/n, Universitat Politecnica de Valencia (UPV), Alcoy, Alicante 03801, Spain

**Keywords:** Gels, Polypropylene films, Rapid method for the quantification of gels in polypropylene films

## Abstract

Polypropylene (PP) films are crucial in various industrial applications, from packaging to medical products. However, a common challenge in PP manufacturing is the presence of gel-like defects. These gels are minor defects on the surface of the films that significantly affect the physicochemical, mechanical, and organoleptic properties of the films, compromising the quality of the final product. This first research focuses on developing and validating an in-line optical method to replace the international method ASTM D 3351–93. The main objective was to create a methodology that has the same scope and analytical performance as those reported by ASTM D 3351–93 in such a way that it can compete with it in terms of precision and accuracy, thus allowing end users to this ASTM, such as PP producers, PP marketers, PP film producers, among others internationally, can use this new methodology with necessary analytical support. This analytical methodology integrates the PP extrusion zones, the film processing stages, and the optical zone for reading and processing analytical data. Additionally, it has the advantage of working with a sample size that is even more representative of the population and has less human error since only one operator is required to carry out the test; this method also has much shorter response times. The developed prototype had 14 online stages that allowed representative quantities of samples to be taken and processed thermally and mechanically for ideal optical measurement. For the online method, a 6-point calibration curve is carried out at concentrations of 40, 10, 5, 2, 1 and 0 ppm for the gel or defect sizes of 200, 400, 500, 600, 700, 800 and 900 µm, showing excellent linearity where the correlation coefficient varied between 0.997 and 0.999, the limits of detection (LOD) varied between 0.85 and 2.61 and the limits of quantification (LOQ) ranged between 2.82 and 8.71. The statistical analyzes by ANOVA of the comparison between the ASTM D 3351–93 method and the proposed simultaneous method indicate that the p value of the evaluation of the means was 0.946, which suggests that the means are not statistically different. To complement, the Tukey test was carried out at 95 %, indicating that the methods have statistical equivalence.

• Process optimization
• Determination of defects or imperfections in PP films

## Graphical abstract

**Figure:**
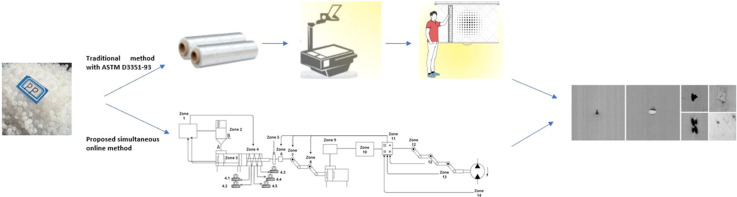

Specifications table

**Table:** 

Subject area	Chemistry
More specific subject area	Analytic chemistry
Name of your method	Rapid method for the quantification of gels in polypropylene films
Name and reference of original method	• Seadrift Polypropylene Plant M-VIII-1. Determination of Gels in PP Film. • ASTM Methods D 3351–93. Teste for Gels Count of Plastic Film.
Resource availability	N.A.

## Method details

### Introduction

**Fig. 1:**
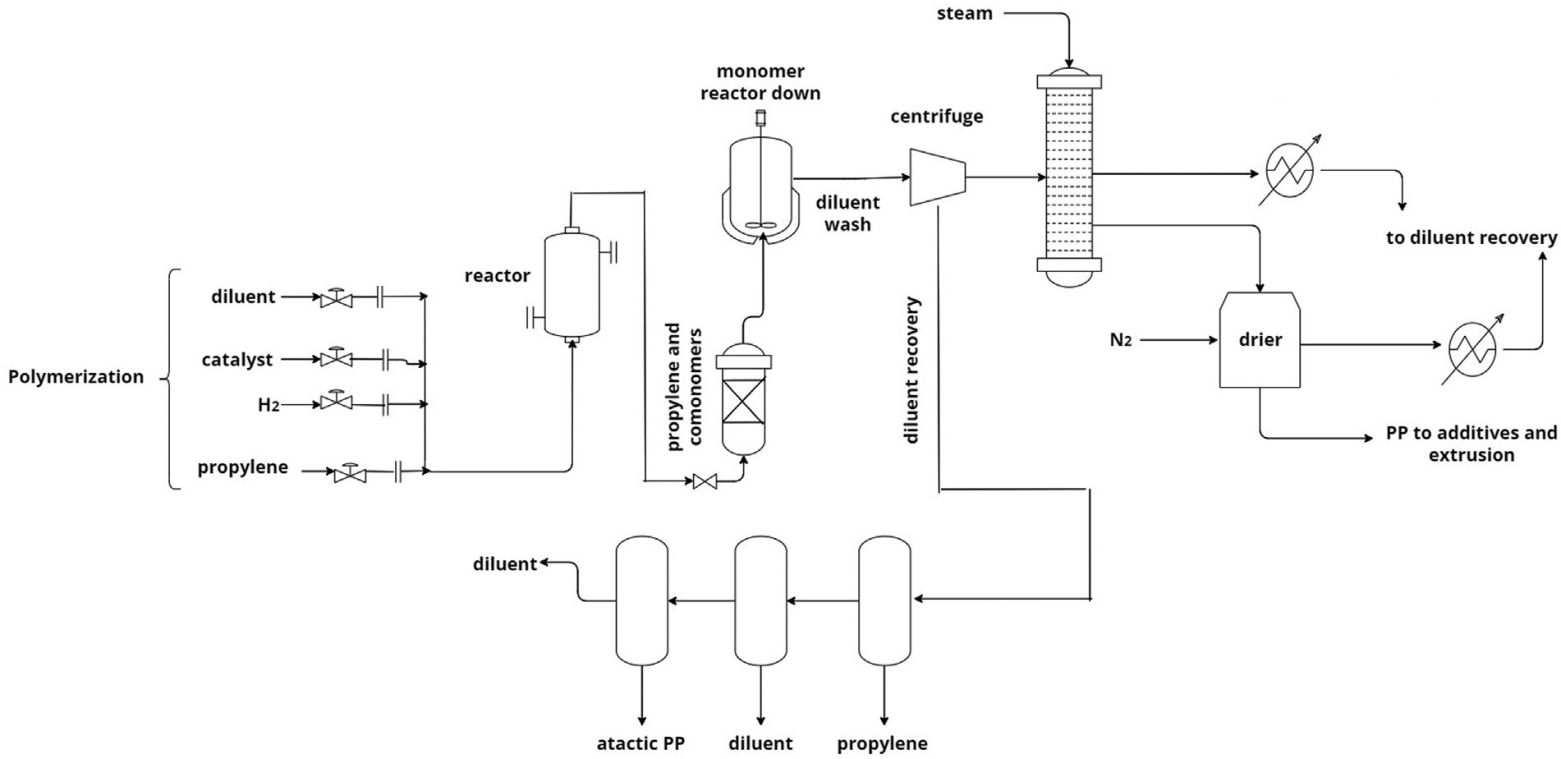
Scheme of the industrial synthesis of polypropylene (PP).

Polypropylene (PP) is an organic polymer from the polyolefin family, industrially synthesized with propylene monomers, an unsaturated aliphatic hydrocarbon with the chemical formula C_3_H_6_ [1,2]. Various raw materials are used in this synthesis process, such as hydrogen, nitrogen, selectivity control agents, and the Ziegler-Natta catalyst [3], as shown in the scheme in Fig. 1. The global relevance of PP lies in its excellent thermal, mechanical, and chemical properties, making it ideal for a wide range of applications, from toys and packaging to automotive parts and clothing [4]. In particular, PP films are crucial as they are thermally and mechanically stretched using techniques in direction. These films exhibit exceptional clarity, low electrostatic charging, puncture resistance, and a high water vapor barrier. In addition, they stand out for their excellent performance in high-speed printing and for not wrinkling or shrinking with environmental changes [5]. All these characteristics make PP films ideal for protecting surfaces during transport and being used as flexible packaging, labels, laminates, and bags [6].

**Fig. 2:**
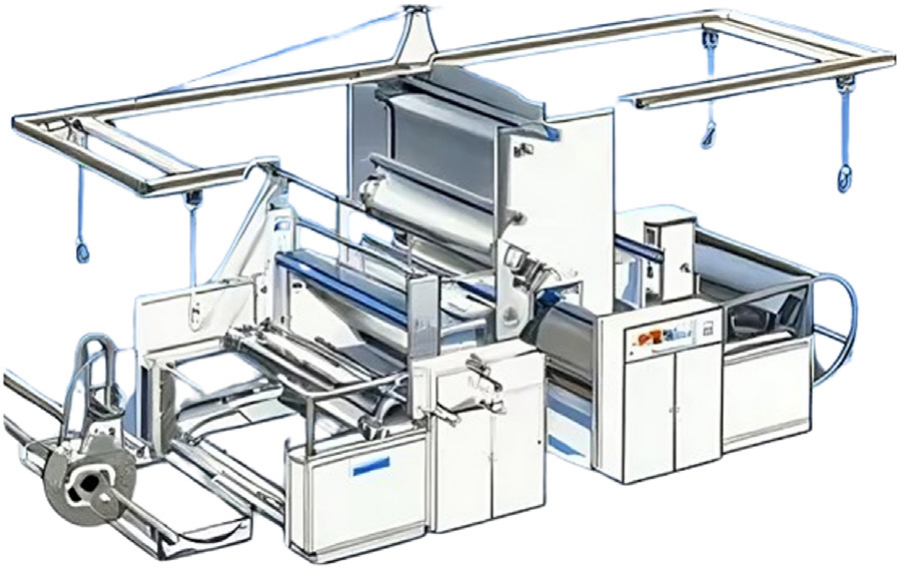
Industrial PP film production scheme.

The high international demand for PP for the applications above indicates a projected growth in the global market, increasing from 86.95 million tons in 2023 to 115.15 million tons by 2028. This increase is estimated at a CAGR of 5.78 % during the forecast period from 2023 to 2028. Within these figures, the significant contribution of PP in the form of films stands out, which, thanks to its exceptional mechanical resistance, superior quality, optical clarity, and flexibility, has become a reliable option for packaging, printing, and lamination applications. The demand for PP in the form of films is projected to exceed 25 million tons in 2023 [7]. PP films are manufactured using extrusion machines composed of a worm screw, five heating zones, a feeding drum, and rollers, as illustrated in Fig. 2 [8]. During the extrusion process of these films, various defects, known as gels, can arise. These defects include black flecks of oxidized PP, cross-linked and oxidized PP, cross-linked PP, unmelted resins, unmelted masterbatches, as well as external contaminants such as metal, insects, wood, dirt, or fabric fibers [[9], [10]]. In this context, a gel is defined as any visible discontinuity in polymer films. A gel may be composed of one or more oxidized, high molecular weight, unfused, unsolved, or cross-linked materials of the same composition as the matrix, which, for various reasons, have not been adequately mixed with the matrix. Gel formation can have multiple sources, such as high molecular weight tails in a bimodal resin, cross-linked material due to overheating, additives with low thermal stability, recycling fine particles, catalyst residues such as silica, and other organic or inorganic contaminations. Due to the shear forces in the extruders, the gels tend to take the shape of elongated ellipses. Those caused by contamination often feature a spot or "fish eye" in the center, while gels caused by high molecular weight material lack this feature. When gels consist of high molecular weight drops, they usually represent primarily an aesthetic problem. However, gels derived from contamination can create a weak spot in a tube or even initiate the formation of a hole in a film [[9], [10]].

**Fig. 3:**
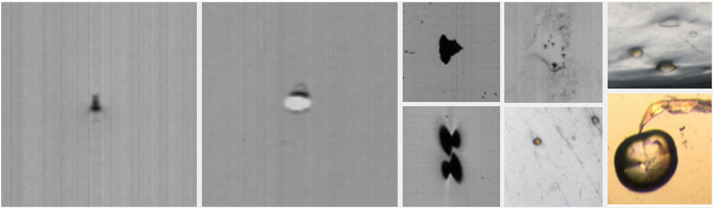
Images of gels or defects between 25 and 200 µm in PP films.

It is essential to determine the origin of gels in an extruded product, as this can indicate whether they come from the initial raw material or were generated during the extrusion process. The gels that form during polymerization are known as P-gels and arise in stagnant areas of the reactor where the resin experiences overheating. Furthermore, these gels can be high molecular weight fractions generated by a bimodal process or by resin produced during reactor maintenance. P-gels are a common problem in polyolefins, and some non-olefin resins can also experience significant problems with gel formation [[10], [11]]. Resin cleanliness represents a critical quality parameter that requires control in polymeric materials intended for high-performance film applications. To evaluate cleanliness in film grade resins, a procedure is commonly followed where a film sample is prepared, and the number of gels present is counted. In addition to the gels originating from the resin, this study also considers external contaminants as gels, such as dirt particles encapsulated in the polymer. Gels can cause problems in thin-walled products such as films, pipes, and fibers, leading to visible defects [11,12]. In thick-walled products, gels are generally invisible and not a problem. Fig. 3 shows the most common appearances of the gels.

Gel quantification is crucial in the plastics industry to evaluate grade resins intended for specific applications. However, no references to gel counting techniques are found in regular journals according to Chemical Abstracts (from 1967 to the present date) and Rubber and Plastics Abstracts (from 1970 to the present date). Within ASTM standards, only one specific test method is available for counting gels in plastic films [13]. The ASTM gel counting method involves placing a film on an overhead projector and measuring the discontinuities visible from the image projected on the screen. However, gels counted in this way are usually larger than 100 μm, with the smallest ones being invisible and difficult to count. An alternative to including smaller gels is to prepare a film sample by stretching it, which reveals colorful cross patterns around each gel under cross-polarization. This method allows the counting of gels by placing the tested sample between sheets of the crossed polarizer and recognizing the voltage concentrators with the naked eye. With the help of the colorful cross patterns, it is possible to count gels as small as ten μm. For even smaller gels, cross-polarized microscopy can be employed on the stretched film to identify smaller stress concentrators that are not discernible to the naked eye. In industrial practice, a specific magnification is chosen based on the minimum gel size that could cause problems in film processing or application. The selected magnification may vary depending on the application and the specific material [14,15].

When a stretched film is observed under cross-polarization, and a gel of considerable size is identified, it is expected to find numerous smaller gels in the vicinity of the larger gel. Analogously, focusing on a smaller gel makes it possible to discover multiple even smaller gels nearby [13]. This self-similarity or self-scaling relationship, present in many other physical systems, is conceptualized by the term "fractal dimension." Mathematically, this gel size distribution can be expressed as:

(1) $$N∼d^{-r_{1}}$$

**Fig. 4:**
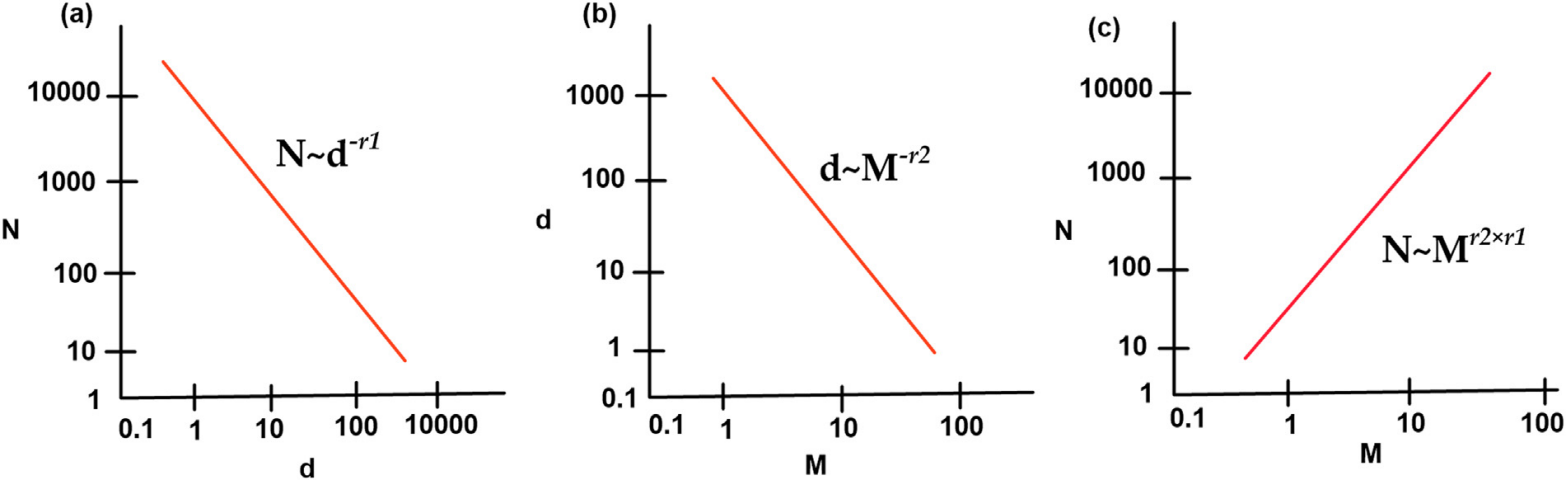
Gel size distribution from gel counts measured at various magnifications; (a) Typical gel size distribution; (b) Smallest size of gels discerned at various magnifications; (c) Gel counts at various magnifications.

In the mathematical expression (1), N is the number of gels per unit volume, d represents the gel size (as the equivalent diameter of the gel), and -r_1_ is an exponent with r_1_ > 0. The inclusion of the negative sign in -r_1_ is used to highlight the inversely proportional relationship between N and d. This distribution is graphically represented in Fig. 4(a).

By examining a film at higher magnification, it is possible to identify gels of smaller dimensions. From a mathematical point of view,

(2) $$d∼M^{-r_{2}}$$

where M is the magnification and -r_2_ is another exponent with r_2_ > 0. Again, the negative sign highlights the inverse proportionality relationship between the gel size (d) and the magnification (M). Fig. 4(b) illustrates this relationship. By substituting Eq. (2) into Eq. (1), we obtain:

$$N∼M^{r_{2}\times r_{1}}\;\left(3\right)$$

Since both r_1_ and r_2_ are positive, the exponent $r_{2}\times r_{1}$ It is also positive. Therefore, the gel count (N) is anticipated to be positively related to the magnification (M), as illustrated in Fig. 4(c). A higher gel count is expected when viewing a film under higher magnification [13]. Every plastic film inevitably contains specific contaminants, either due to the production process or the inherent characteristics of the polymers. The presence of gels in this context becomes a matter of resolution. In extrusion and converting lines, we encounter various defects, such as gels, black specks, holes, oil stains, and air bubbles. Although these problems have different origins, it is essential to highlight that most can hurt subsequent processing [13]. Counting ASTM gels at a single magnification does not provide complete information about gel size distribution, as it only provides a snapshot at a particular scale without capturing the entire image. Even advanced image analyzers that directly measure gel size cannot obtain the full gel size distribution at a single magnification. Regardless of the magnification used, based on the gel size distribution shown in Fig. 4(a), much of the measurement time would be spent on small gels, making it impossible to measure enough statistically. Removing these defects from extruded polypropylene film products can be difficult, time-consuming, and costly due to the complexity of the problem and the product falling outside of the required specifications. To guarantee product quality, an advanced optical inspection system is needed. These systems identify and locate all defects, capture precise images of their position, and alert the operator in real-time. This research aims to provide an improved method for identifying gel types in polypropylene films. The benefits of incorporating the proposed method include improvements in the film extrusion and converting process, more efficient management of incoming raw material, and modification of the final product, especially during the converting process. Providing immediate feedback on the presence of gels and black specks reduces machine downtime for die cleaning while optimizing machine capacity. In the extrusion and converting process, a web inspection system can monitor raw material quality and facilitate the selection of the best combination of materials.

### Methods

#### Scheme of classic analyses in the laboratory

**Fig. 5:**
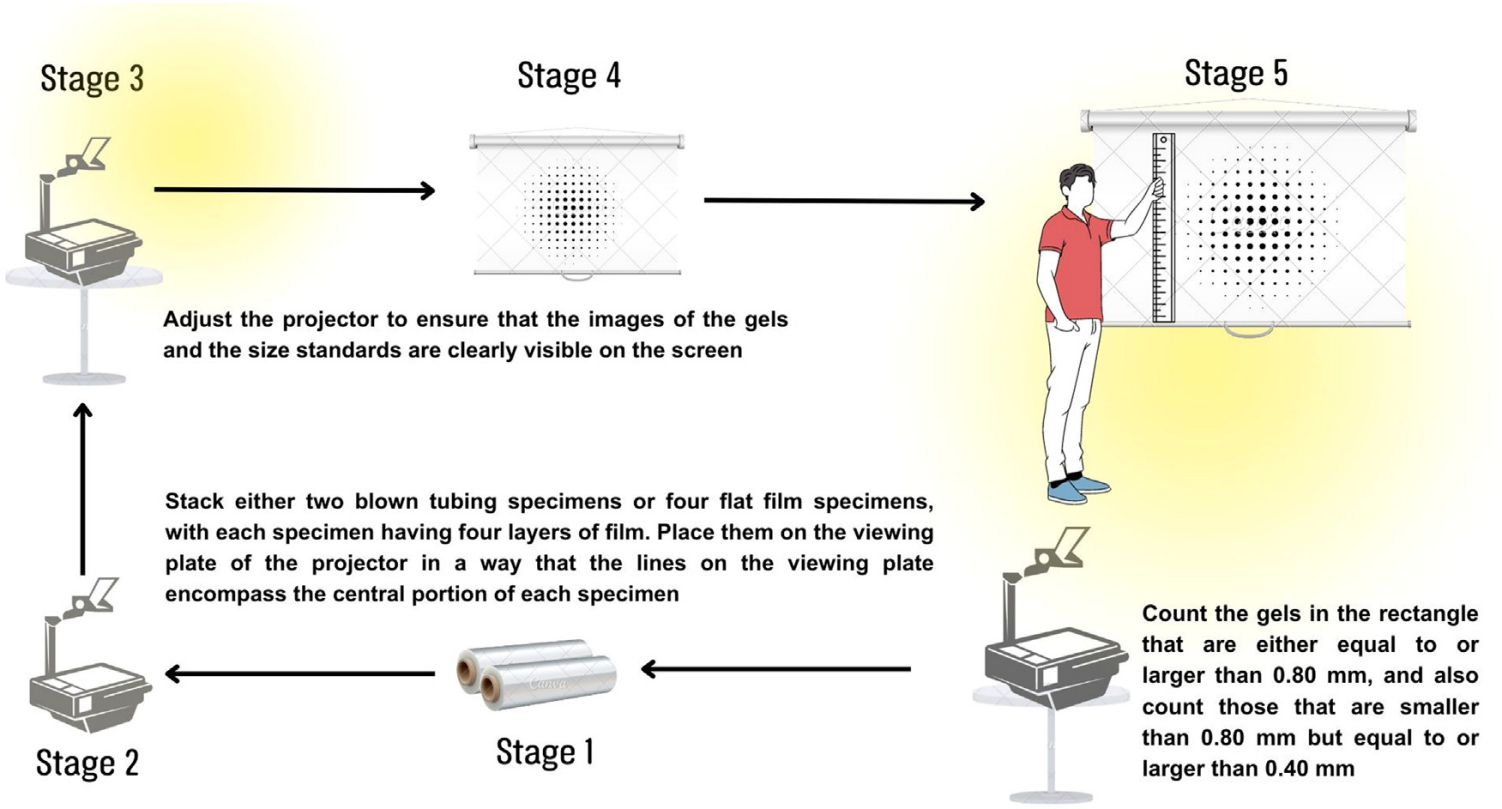
General scheme of laboratory-scale analysis which ASTM D3351–93.

The conventional method based on the ASTM D3351–93 standard for the traditional analysis of defects or the quantification of gels larger than 100 µm in polypropylene plastic films involves the observation of images generated by projecting a PP film using light on projector equipment. Table, as illustrated in Fig. 5, where five important steps are evident. It is a requirement that the projected film meets technical thickness specifications of 100 µm or less. This approach was developed in response to the difficulties and fatigue associated with manual gel counting, offering a method with significant limitations. These limitations are mainly related to the size, quantity, and representativeness of the samples, in addition to the inherent subjectivity of each operator during the execution of the test.

This method requires specific equipment, such as an overhead projector with a viewing plate of at least 200×200 mm and a projection screen spanning at least 2 × 2 m. Additionally, an acrylic plate measuring approximately 230×230 mm with a thickness of 3.2 mm is required, which includes two standard holes, one with a diameter of 0.80 mm and another with a diameter of 0.40 mm. In preparing the specimens and performing measurements, four layers of film, each with a minimum area of 200 m^2^m, are cut and assembled in a corner, and three test specimens are created. The inspection covers an area of 190×200 mm across the four film layers, resulting in a total test area of 1520 cm^2^ [15].

Within the context of the regulatory procedure established by ASTM (American Society for Testing and Materials), the projector and screen are configured to achieve a magnification ranging from 8 to 1 to 10 to 1. This is done with care to avoid any distortion in the projection by ensuring that the line from the mirror to the screen is perpendicular. Two blown tube samples or four flat film samples are placed on the projector viewing plate. Each sample comprises four overlapping layers of foil, and it is ensured that the lines on the display plate cover the middle part of each sample. The transparent plastic plate with standard holes is placed in contact with the stack of samples, applying pressure to minimize wrinkling and preventing the staple that holds the film layers together from interfering between the plates. The projector is then focused so that the images of the gels and gel size standards are projected clearly on the screen. In the counting process, gels within the rectangle are considered, classifying them as those as large as or more significant than the 0.80 mm standard (referred to as most significant), as well as gels smaller than the 0.80 standard. mm but as large as or more important than the standard 0.40 mm (referred to as more minor). This procedure conforms to the regulations established, specifically ASTM D3351–93, for analyzing defects in PP plastic films. [15,16].

#### Prototype of the systematization of the simultaneous analysis

**Fig. 6:**
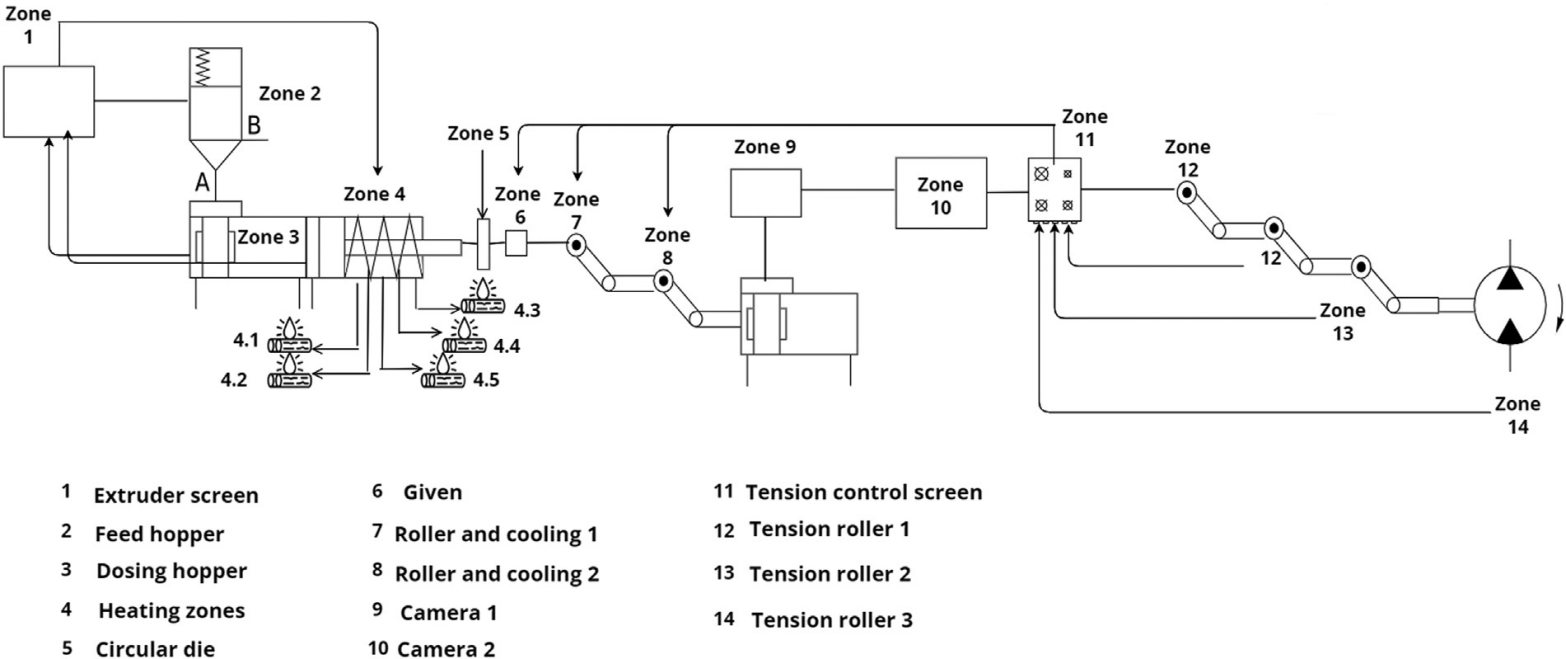
General prototype scheme of the simultaneous analysis.

Using the procedure established on a laboratory scale, a prototype is designed for the systematization of the analysis through the semi-automation of shown in Fig. 6 and the table of gel counting events is shown in Table 1

**Table 1:** Table of valve events of the simultaneous analysis prototype.

Valve	Time on (min)	Comments
Zone 1	0.5	Extruder screen
Zone 2	1.0	Feed Hopper
Zone 3	1.5	Dosing Hopper
Zone 4.1	2.0	Heating zone 190 °C
Zone 4.2	2.5	Heating zone 195 °C
Zone 4.3	3.0	Heating zone 220 °C
Zone 4.4	3.5	Heating zone 210 °C
Zone 4.5	4.0	Heating zone 220 °C
Zone 5	4.5	Cast outlet nozzle
Zone 6	5.0	Extruder Die
Zone 7	5.7	Roller and cooling 1
Zone 8	6.4	Roller and cooling 2
Zone 9	6.5	Camera 1
Zone 10	7.0	Camera 2
Zone 11	7.2	Tension control screen
Zone 12	7.5	Tension roller 1
Zone 13	7.6	Tension roller 2
Zone 14	7.8	Tension roller 3

The prototype can carry out measurements of optical and physical properties in the production of high-quality cast films. This process includes extrusion, cooling, stripping, and winding stages to obtain flat films. All settings and parameters, such as extruder speed, temperature, film tension, winding force, and winder diameter, are recorded through a touch panel control system. This ensures that the quality of the movie can be played at any time. This aspect is crucial for performing online and offline optical and physical measurements, encompassing the detection of gels, contaminations, degradations, and other impurities.

The system is composed of an optoelectronic module (11) for inspecting polymer films, being versatile for its application in laboratories and ongoing production processes. Film inspection is performed using a high-resolution linear camera and a high-power LED. This configuration enables optimal detection of defects in transparent, opaque, and colored polymer films. The measurement results are analyzed by software that adapts to the user's specific needs, classifying defects and determining the quality of the film. Additionally, this system can be integrated with other film inspection devices, such as an X-ray tape analyzer, to obtain additional measurements and allow for a more detailed analysis of defects.

## Method validation

### Validation of ASTM D3351–93 method vs simultaneous online method

Taking into account the proposed approach, which involves the creation of a prototype capable of carrying out various tests simultaneously, we characterized a sample of films from the same batch, using the same operations, to analyze the content of gels and impurities present.

### Linearity

Our study evaluated the linearity of the proposed method for counting gels in PP plastic films as an alternative to the traditional method based on the ASTM D3351–93 standard. This analysis focused on different gel sizes expressed in microns and their concentrations in parts per million (ppm). Concentrations of 40, 10, 5, 2, and 1 ppm of standards of size 200, 400, 500, 600, 700, 800, and 900 μm are prepared. For each concentration range of interest, analytical performance is evaluated. The results in Table 2 revealed a strong linear relationship between the concentration of gels and the counts obtained, supported by high coefficients of determination (R²) in all conditions evaluated. The linearity of the method is essential for its practical application, as it ensures that measurements are proportional and predictable over a specific range of concentrations. In our case, the ability of the method to provide consistent linear responses across different gel sizes suggests its reliability and robustness. Implementation of this method offers several significant advantages compared to the conventional approach. First, it eliminates the subjectivity associated with visual gel counting, providing a more precise and objective quantitative measurement. Furthermore, by providing a linear evaluation, the method facilitates interpreting and comparing results under different conditions and gel sizes. The linearity of the proposed method also suggests its applicability to various gel concentrations, allowing its use in multiple situations and applications. This versatility is essential to address different production scenarios and ensure accurate and consistent evaluation of the presence of gels in PP films.

**Table 2:** Determination of linearity.

Microns	ppm	Count	LOD (μm)	LOQ (μm)	Equation	R^2^
200	40	945	2.61	8.71	*y* = 23.719x – 7.2802	0.999
	10	210				
	5	124				
	2	38				
	1	15				
	0	0				
400	40	724	1.81	6.03	*y* = 18.245x – 9.3726	0.9992
	10	159				
	5	81				
	2	28				
	1	10				
	0	0				
500	40	610	2.39	7.99	15.267x – 5.2514	0.9981
	10	127				
	5	72				
	2	33				
	1	12				
	0	0				
600	40	512	0.85	2.82	12.932x – 5.8469	0.9997
	10	121				
	5	61				
	2	18				
	1	3				
	0	0				
700	40	475	1.78	5.92	*y* = 12.124x – 9.8626	0.9983
	10	118				
	5	41				
	2	7				
	1	3				
	0	0				
800	40	300	1.57	5.23	*y* = 7.6351x – 3.3062	0.9967
	10	85				
	5	31				
	2	5				
	1	2				
	0	0				
900	40	324	1.42	4.74	*y* = 8.3039x – 8.7708	0.9977
	10	77				
	5	24				
	2	3				
	1	1				
	40	324				

### Precision and accuracy

Precision was measured regarding repeatability (intraday precision), represented as a relative standard deviation. Five replicates were carried out for each sample in both methods using the same operator, varying the measurement range, as indicated in Table 3, to determine the repeatability (RPED) of the process. Each laboratory and prototype operation was carried out on the same day by the same operator using the same instrument. Various researchers have established acceptance criteria for precision and/or accuracy (bias). It is stipulated that precision should be within 15 % of RSD, with the exception of the LLOQ where up to 20 % of RSD is accepted. Likewise, bias is required to be within ±15 % of the expected true value, except for the LLOQ where a margin of ±20 % is allowed [17,18].

**Table 3:** Precision and accuracy in laboratory and prototype results.

Intraday Test - ASTM D3351–93 Method Vs Simultaneous Online Method										
Replicas	Measuring range (µm)	µm	ASTM Defect Count	Online defect counting	ASTM Average	Online average	ASTM deviation	Online deviation	RSD ASTM	RSD on line
1	400–800	400	55	56	57	57	2739	2121	4,8	3,7
2	400–800	400	60	59						
3	400–800	400	55	54						
4	400–800	400	60	59						
5	400–800	400	55	57						
1	400–800	500	58	59	61	60,6	2915	2074	4,8	3,4
2	400–800	500	60	62						
3	400–800	500	65	63						
4	400–800	500	63	61						
5	400–800	500	59	58						
1	400–800	600	60	61	64,6	64,6	2966	2191	4,6	3,4
2	400–800	600	66	65						
3	400–800	600	64	65						
4	400–800	600	68	67						
5	400–800	600	65	65						
1	400–800	700	65	64	64,4	63,4	2302	2302	3,6	3,6
2	400–800	700	62	61						
3	400–800	700	68	67						
4	400–800	700	63	62						
5	400–800	700	64	63						
1	400–800	800	68	69	71,8	71,6	2864	2408	4,0	3,4
2	400–800	800	74	73						
3	400–800	800	72	73						
4	400–800	800	70	69						
5	400–800	800	75	74						
1	>800	900	103	104	105,8	105,2	3962	2588	3,7	2,5
2	>800	900	106	105						
3	>800	900	108	106						
4	>800	900	101	102						
5	>800	900	111	109						
1	>150	200	55	57	58,6	59,2	2302	1483	3,9	2,5
2	>150	200	58	59						
3	>150	200	59	60						
4	>150	200	60	59						
5	>150	200	61	61						

The results obtained from the comparison between the ASTM Method and the simultaneous online Method in different measurement ranges (µm) indicate a notable consistency in counting defects in plastic films. In the 400–800 µm range, both methods present consistent defect counts, demonstrating agreement in imperfection detection. This trend remains in the ranges greater than 800 µm and greater than 150–200 µm, where defect counts between methods are comparable. The similarity in the results suggests a consistent effectiveness of both the ASTM Method and the simultaneous online Method in the evaluation of the quality of plastic films, supporting the validity and usefulness of the simultaneous online Method as an effective alternative in the detection of defects. In the measurement range of 400–800 µm, a notable similarity was evident in the averages obtained between the ASTM Method and the simultaneous online Method, indicating agreement in the results. Going deeper into the assessment of intraday precision, relative standard deviation (RSD) emerges as a crucial indicator. The standard deviations and RSDs in this range remained comparable between both methods, within an acceptable range of 4.8 % to 3.7 %. When considering the measurement range greater than 800 µm, the closeness between the averages of both methods was again highlighted. However, the online simultaneous method tended to present lower standard deviations and RSDs (2.588 % to 3.962 %, 2.5 % to 3.7 %, respectively) compared to the ASTM Method, suggesting greater consistency and stability in measurements. The lower standard deviation and RSD in the online simultaneous method indicate that the measurements tend to be more consistent and stable, reflecting more excellent reliability and precision in counting gels in plastic films. This strengthens the validity and usefulness of the Simultaneous Online Method as an effective alternative to the ASTM Method. In the measurement range greater than 150–200 µm, the previous trend was corroborated, reaffirming that the simultaneous online Method tends to offer lower standard deviations and RSD than the ASTM Method. Meeting acceptability criteria, where average values and RSDs are maintained at levels that verify within-day precision for both methods, adds robustness to the evaluation. Furthermore, the divergence from the predicted value, which remains below 15 %, supports the claim that both methods meet acceptable intraday accuracy criteria.

**Fig. 7:**
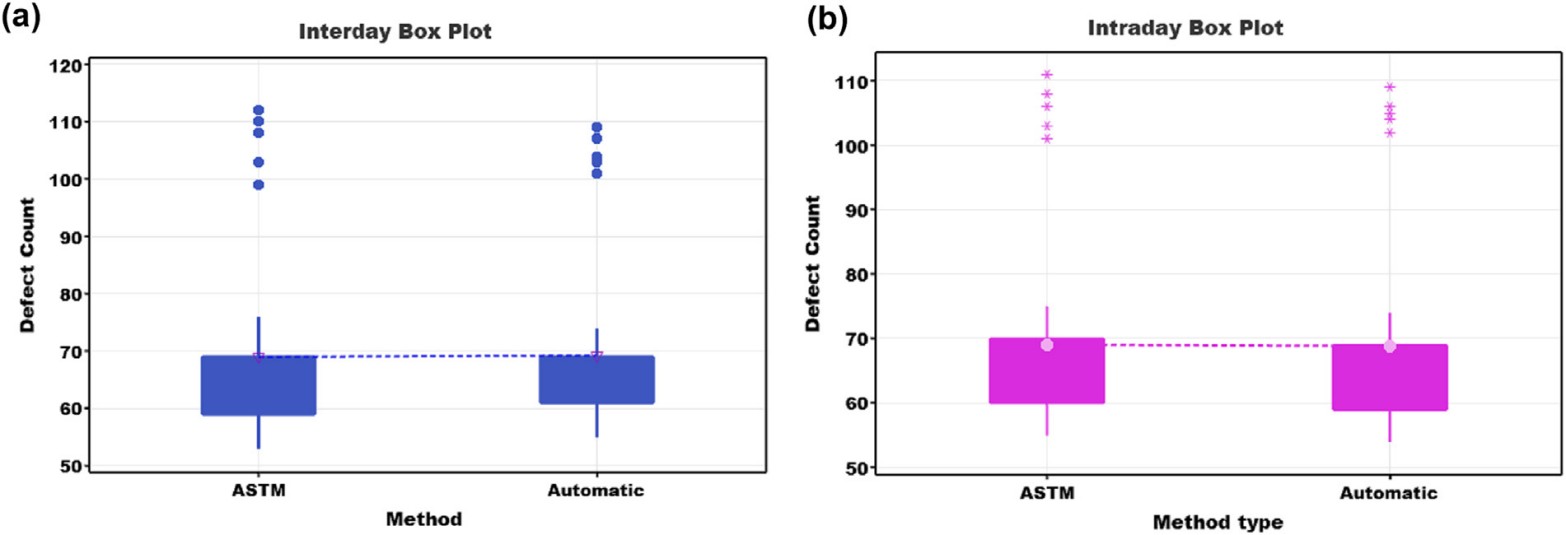
95 % confidence intervals. a) Box diagram of interday tests: laboratory tests versus prototype tests. b) boxplot of intraday testing: laboratory testing versus prototype testing.

In the present study, the analysis of variance (ANOVA) was implemented to determine the statistical significance of the possible differences between the means of the data (See Fig. 7a, b and Table 4). The ANOVA test was used to compare the p-value with a significance level, denoted as α (alpha), usually set at 0.05. A significance level of 0.05 implies a 5 % risk of concluding that there is a difference when, in fact, there is none. For values of *p* ≤ α, some of the means are considered statistically significant, while for values of *p* > α, the differences between the means do not reach statistical significance.

**Table 4:** One-way ANOVA: laboratory value; prototype value.

Variance analysis					
Source	GL	Adj SC	Adj MC.	F-Value	P-Value
Factor	1	1,2	1157	0,00	0,946
Error	68	17,265,5	253,904		
Total	69	17,266,6			
Means					
Factor	N	Mean	Std. Dev.	IC de 95 %	
ASTM	35	68,94	16,44	(63,57; 74,32)	
Automatic	35	69,20	15,41	(63,83; 74,57)	
Tukey Method and 95 % Confidence					
Factor	N	Mean	Grouping		
Automatic	35	69,20	A		
ASTM	35	68,94	A		

In the analysis of variance (ANOVA), the statistical significance of the differences between the means of the two methods, 'ASTM' (reference method) and 'Automatic' (proposed method), was evaluated for the measurement of specific parameters. The null hypothesis stated that the population means of both methods are the same, while the alternative hypothesis suggests that at least one of the means is different. The results obtained from the ANOVA are summarized in Table 4.

Comparison of the p-values with the significance level α (0.05) revealed that the p-value (0.946) is more significant than α, indicating insufficient evidence to reject the null hypothesis. This suggests no statistically significant differences exist between the population means of the 'ASTM' and 'Automatic' methods. Therefore, the results support the acceptance of the proposed method as an effective alternative to the reference method ('ASTM') for measuring the considered parameters. The means and overlapping confidence intervals further support the conclusion that the methods have no statistically significant differences.

**Fig. 8:**
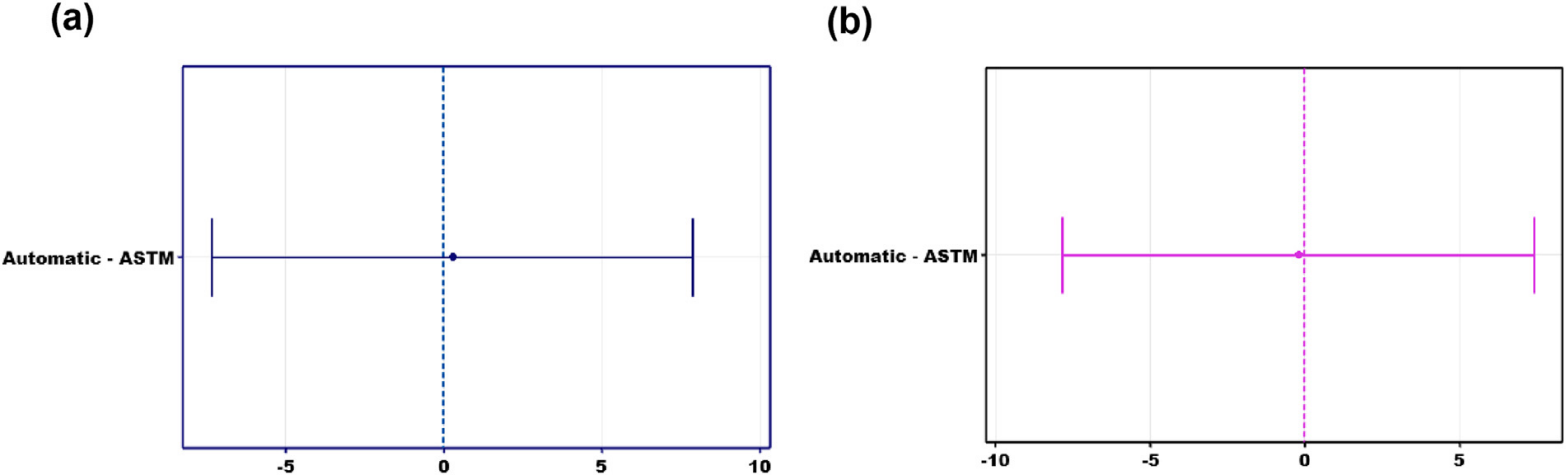
Tukey simultaneous 95 % Cis. Difference of means for laboratory and prototype. a) interday tests b) intraday tests.

Additionally, the Tukey method in ANOVA was applied, as detailed in Fig. 8a and b. The Tukey analysis was implemented to establish confidence intervals that assess the differences between the means of the factors, controlling the error rate per family at a specific level. The observation of the letter A shared among the values of the ASTM method and the prototype reflects statistical equivalence. Furthermore, Fig. 8 illustrates that the interval of interest approaches zero, indicating the absence of statistically significant differences. These results strengthen the validity and consistency of the conclusions obtained in both configurations, supporting the suitability of the proposed prototype as a reliable alternative in the mentioned measurements.

## Conclusion

This method makes it possible to automate specific tests to evaluate the presence of gels in films at an industrial level. This implementation reduces analysis time and facilitates immediate adjustments in process controls, avoiding the generation of products that need to meet established quality parameters. The model proposal is presented as an optimal alternative for automating the characterizations carried out on polypropylene (PP) samples, allowing not only the execution of a systemic and simultaneous process but also a reduction in operation times. This approach especially benefits the last steps in producing films or other PP-based products, thus optimizing the efficiency and quality of the process.

## Ethics statements

Not applicable.

## CRediT authorship contribution statement

**Hernández-Fernández Joaquin:** Conceptualization, Methodology, Software, Data curation, Writing – original draft, Visualization, Investigation, Supervision, Validation, Writing – review & editing. **Ortiz Katherine:** Data curation, Writing – original draft, Supervision, Software, Validation, Writing – review & editing. **Lopez-Martinez Juan:** Data curation, Writing – original draft, Visualization, Investigation, Writing – review & editing.

## Declaration of competing interest

The authors declare that they have no known competing financial interests or personal relationships that could have appeared to influence the work reported in this paper.

## Data Availability

Data will be made available on request.
